# Food texture affects glucose tolerance by altering pancreatic β-cell function in mice consuming high-fructose corn syrup

**DOI:** 10.1371/journal.pone.0233797

**Published:** 2020-05-29

**Authors:** Naoki Harada, Masayuki Nomura, Yasuhiro Yoda, Shigenobu Matsumura, Hiroshi Inui, Ryoichi Yamaji

**Affiliations:** 1 Division of Applied Life Sciences, Graduate School of Life and Environmental Sciences, Osaka Prefecture University, Sakai, Osaka, Japan; 2 Division of Food Science and Biotechnology, Graduate School of Agriculture, Kyoto University, Uji, Kyoto, Japan; 3 Department of Nutrition, College of Health and Human Sciences, Osaka Prefecture University, Habikino, Osaka, Japan; Max Delbruck Centrum fur Molekulare Medizin Berlin Buch, GERMANY

## Abstract

The incidence of metabolic diseases, such as type 2 diabetes, has increased steadily worldwide. Diet, beverages, and food texture can all markedly influence these metabolic disorders. However, the combined effects of food texture and beverages on energy metabolism remains unclear. In the present study, we examined the effect of food texture on energy metabolism in mice administered high-fructose corn syrup (HFCS). Mice were fed a soft or hard diet along with 4.2% HFCS or tap water. Body weight and total caloric intake were not affected by food texture irrespective of HFCS consumption. However, caloric intake from HFCS (*i*.*e*., drinking volume) and diet were higher and lower, respectively, in the hard food group than in the soft food group. The hard food group’s preference for HFCS was absent in case of mice treated with the μ-opioid receptor antagonist naltrexone. Despite increased HFCS consumption, blood glucose levels were lower in the hard-diet group than in the soft-diet group. In HFCS-fed mice, insulin levels after glucose stimulation and insulin content in the pancreas were higher in the hard food group than the soft food group, whereas insulin tolerance did not differ between the groups. These food texture-induced differences in glucose tolerance were not observed in mice fed tap water. Thus, food texture appears to affect glucose tolerance by influencing pancreatic β-cell function in HFCS-fed mice. These data shed light on the combined effects of eating habits and food texture on human health.

## Introduction

The prevalence of metabolic diseases, such as obesity and type 2 diabetes mellitus (T2DM), has been increasing steadily in both developed and developing countries [1, 2]. This increased incidence is related to diet and lifestyle changes, rather than genetic factors. Increased caloric intake beginning from the 1970s, is highly associated with obesity in the United States [3]. T2DM, a disorder of glucose metabolism, is closely associated with obesity in people in Western countries, but occurs less frequently in people in Asian countries [1]. T2DM develops from the relative lack of insulin due to both insufficient secretion of insulin from pancreatic β-cells (particularly prevalent in people in Asian countries) and insulin sensitivity in peripheral tissues such as muscle, liver, and adipocyte tissue (particularly prevalent in people in Western countries). Environmental factors strongly influencing T2DM prevalence include dietary habits.

The texture of the daily diet (*i*.*e*., if the food is hard or soft) affects chewing habits and is important for physical and mental health as well as energy metabolism [4, 5]. Consumption of a soft diet is thought to lead to overeating and obesity [6] and the development of T2DM [7], however, these findings are somewhat controversial [5–8]. These discrepancies may be attributed to additional food or prandial components; mastication of food, for example, can affect brain function [9]. A range of different behaviours may therefore greatly affect these findings. β-Endorphin, an opioid neuropeptide, stimulates the μ-opioid receptor and affects behaviours such as food intake by influencing the reward system [10].

High-fructose corn syrup (HFCS) is a liquid sweetener composed of fructose and glucose that is widely used in beverages [3]. HFCS intake can affect the brain reward system [11]. Compared to sucrose, HFCS is advantageous for food manufacturers due to its enhanced stability and solubility [12]. Use of HFCS increased from the 1970s to the 1990s [3], preceding an explosive increase in the incidence of metabolic diseases, including obesity and T2DM. Although increased HFCS consumption promotes caloric intake and is postulated to underlie the increased recent incidence of metabolic diseases [13, 14], this connection remains controversial because of the lack of robust and direct evidence [3]. It is also unclear whether moderate doses of HFCS promotes metabolic disease through mechanisms independent of increased caloric intake.

The management of energy metabolism is important for the prevention of lifestyle-related metabolic diseases. Dietary habits are complex, and the interactive effects of food texture and HFCS still remain unclear. In the present study, we studied the effects of consuming food with different texture (hard or soft) on the preference for HFCS intake in mice. Furthermore, we investigated the effects of the daily diet texture on glycaemic control in mice administered HFCS in their water.

## Materials and methods

### Animals

Hard or soft food was prepared using standard chow (CE2, 3.44 kcal/g, CLEA Japan, Tokyo, Japan). The hard food was prepared by autoclaving standard chow to increase hardness [15]; while the soft food was prepared by pulverising the hard food with a mixer, followed by passage through a sieve. Both food textures had the same nutritional composition. Male 3-week-old C57BL/6J mice (10–13 g) were obtained from Kiwa Laboratory Animals (Wakayama, Japan). Mice were individually housed under conventional conditions with controlled temperature (23 ± 3°C) and lighting (12 h light and 12 h dark cycle, lighting period starting at 08:00 h) conditions, with *ad libitum* access to food and water. After habituation with the soft food for 1 week, mice were separated into groups randomly, so that each group had a similar body weight distribution. Drinking water containing HFCS (4.2%, w/v, 0.16 kcal/ml) was prepared by mixing fructose: glucose = 2.3% (w/v): 1.9% (w/v) [16]. The ratio of fructose and glucose is based on HFCS-55, which is composed of 55% fructose and 45% glucose [3]. Water containing HFCS was replenished once a week. Body weight, dietary intake, and drinking water were measured weekly. To perform an intraperitoneal glucose tolerance test (IPGTT) and insulin tolerance test (ITT), mice were fasted for 6 h (from 09:00), and then, 2 g/kg glucose or 1 U bovine insulin were injected intraperitoneally. Blood was collected from the tail vein and glucose levels were measured using a Stat Strip Express Glucose/Ketone meter (Nova Biomedical, Tokyo, Japan). For measuring insulin levels, mice were fasted for 6 h (from 09:00), and then, glucose (3 g/kg, *i*.*p*.) was injected. Blood was collected from the facial vein at 15 min after glucose injection. Plasma insulin levels were measured using the mouse insulin ELISA Kit (Shibayagi, Gunma, Japan). At 21 weeks of age, after 4 h of fasting, the mice were put under deep general anaesthesia using a mixture of three drugs administered by intraperitoneal injection: 0.3 mg/kg medetomidine hydrochloride (Nippon Zenyaku Kogyo, Tokyo, Japan), 4.0 mg/kg midazolam (Fuji Pharma, Tokyo, Japan), and 5.0 mg/kg butorphanol tartrate (Meiji Seika Pharma, Tokyo, Japan); then, they were sacrificed by complete blood collection via the inferior vena cava, and the plasma and organs were collected for further analysis. Naltrexon (10 mg/kg, *s*.*c*.) was injected twice (08:00 and 20:00)/day for 7 days, after 2 day of saline treatment (*s*.*c*.) in 5-week-old mice. All animal experiments were approved by the Animal Care and Use Committee of Osaka Prefecture University (Nos. 29–192, 30–137, 19–43, and 19–155) and were performed in compliance with its guidelines.

### Immunohistochemistry

Tissue staining was performed as described previously [17]. Briefly, the pancreatic tail was fixed and embedded in paraffin. Pancreatic tissue sections from the middle of the tissue were sectioned at a thickness of 4 μm. Pancreatic β-cells were stained with mouse anti-insulin antibody (1:10,000, D6C4, Hytest, Turku, Finland), Histofine Simple Stain (Nichirei, Tokyo, Japan), and 3,3’-diaminobenzidine substrate kit for peroxidase (Vector Laboratories, Burlingame, CA, USA). Then, tissue sections were counterstained with haematoxylin. Tissue sections were observed under a light microscope (BZ9000, Keyence, Osaka, Japan). Densitometric measurements were performed using ImageJ software version 1.4.3.67 (National Institutes of Health, Bethesda, MD, USA).

### Measurement of pancreatic insulin contents

Pancreatic tails were homogenised in acid-ethanol (0.18 M HCl in 70% ethanol), followed by neutralisation with 1 M Tris-HCl, pH 7.5. The solutions were diluted with PBS containing 0.05% Tween-20, and insulin concentrations were determined by ELISA as described previously [18].

### Measurement of plasma and liver lipid, urinary creatinine, and adiponectin levels

Lipids from the liver were extracted using the Folch method [19], with minor modifications. Triglyceride and cholesterol levels in the liver and plasma were determined using the triglyceride E-Test or cholesterol E-Test (FUJIFILM Wako Pure Chemical Corp., Osaka, Japan). Urine was collected from mice at 17 weeks of age. Urine protein concentrations were determined using the Bradford assay [20] and normalised to urine creatinine concentrations, determined using the commercially available kit, LabAssay Creatinine (FUJIFILM Wako Pure Chemical Corp., Osaka, Japan). Plasma adiponectin levels were determined as described previously [21].

### Quantitative PCR

Total RNA was isolated from the tissues using Sepasol-RNA I super G (Nacalai Tesque, Kyoto, Japan). After treatment with DNase I, total RNA was reverse transcribed to cDNA with ReverTra Ace (TOYOBO, Osaka, Japan) and the oligo(dT)20 primer. Transcribed cDNA was used for quantitative PCR analysis using TB Green Premix *Ex Taq* II DNA polymerase (Takara Bio, Shiga, Japan) at an annealing temperature of 55–60°C on a Thermal Cycler Dice TP850 (Takara Bio). Specific primers for target genes were as follows: *phosphoenolpyruvate carboxykinase* (*Pepck*), sense 5′-GGTGTTTACTGGGAAGGCATC-3′ and antisense 5′-CAATAAGGGGCACTGGCTG-3′; *pyruvate carboxylase* (*Pc*), sense 5′-GAGCTTATCCCGAACATCCC-3′ and antisense 5′-TCCATACCATTCTCTTTGGCC-3′; *insulin degrading enzyme* (*Ide*), sense 5′-TGGTCATCTAATTGGGCACG-3′ and antisense 5′-AACCTCGGGCTCCTTCCTTC-3′; *fibroblast growth factor 21* (*Fgf21*), sense 5′-GCTGCTGGAGGACGGTTACA-3′ and antisense 5′-CACAGGTCCCCAGGATGTTG-3′; or β-actin, sense: 5′-TTGCTGACAGGATGCAGAAG-3′ and 5′-GTACTTGCGCTCAGGAGGAG-3′. The relative expression levels of the target genes were calculated by the standard curve method using Ct values. The specificity of each PCR product was confirmed by electrophoresis and dissociation curve analysis.

### Statistical analysis

Data were analysed by *t*-test or two-way analysis of variance (ANOVA) with Tukey-Kramer’s post-hoc test, using JMP statistical software version 8.0.1 (SAS Institute, Cary, NC, USA). Data are shown as the means ± standard errors (SEs), and *p* < 0.05 was considered to be statistically significant.

## Results

### The ratio of energy intake from HFCS to total energy intake in mice fed with different concentrations of HFCS

**Fig 1:**
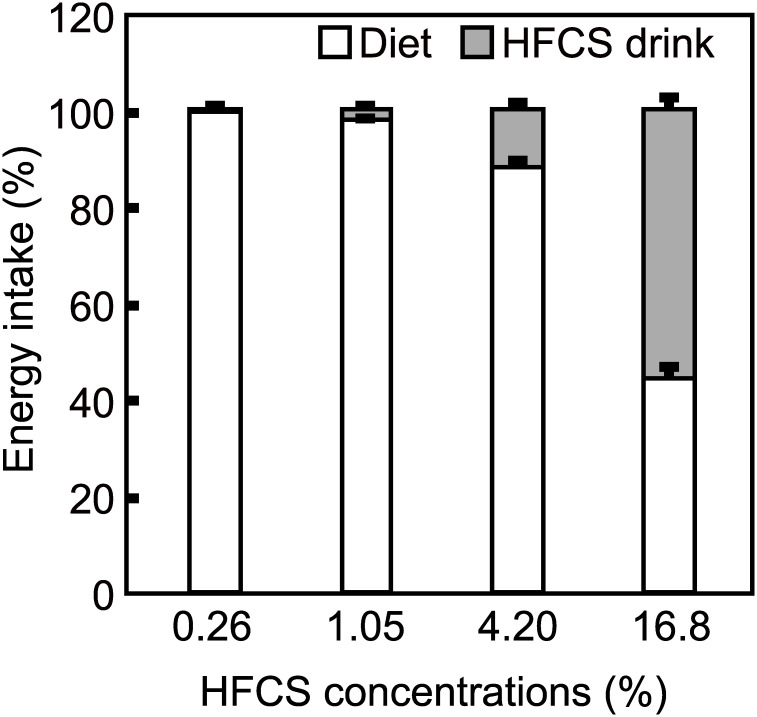
Relationship between high-fructose corn syrup (HFCS) concentration and contribution of HFCS to total energy intake. Mice were administered increasing concentrations of HFCS, receiving 0.26, 1.05, 4.2, 16.8% HFCS between 4 and 7 weeks, respectively. The percent total energy from HFCS was calculated. Data are expressed as the means ± SEs (n = 6).

Mice fed with soft food were provided HFCS at different concentrations for 1 week. The ratio of energy intake from HFCS water to that from solid food was calculated. The ratio of energy intake from HFCS increased in parallel with an increase in HFCS concentration (Fig 1). The mean contribution of HFCS to total energy intake was 0.3, 1.7, 12, and 56% in mice receiving 0.26, 1.05, 4.2, and 16.8% HFCS, respectively. Based on these results, 4.2% HFCS was selected for use in long-term breeding studies because the energy contribution from this concentration of HFCS in mice is similar to the level typically ingested by people in the United States (~15% calories from HFCS and/or sucrose) [3, 22].

### Body weight and energy intake of mice fed hard or soft food with access to tap water or HFCS in long-term breeding

**Fig 2:**
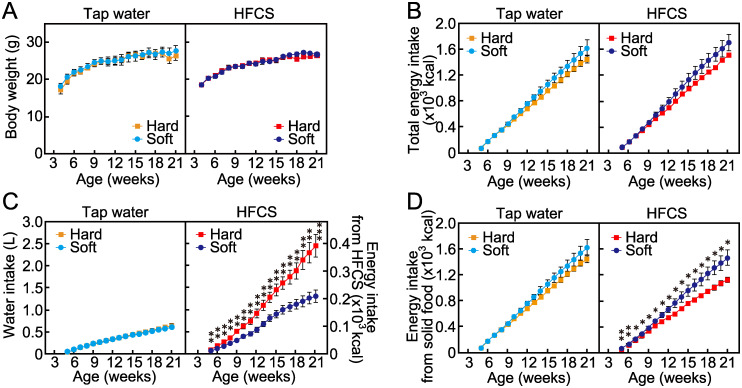
Effect of food texture on body weight and food or drink intake of mice. (A) Body weight, (B) cumulative total calorie intake, (C) cumulative water intake, and (D) cumulative calorie intake from solid food of mice fed hard or soft food, with or without 4.2% high-fructose corn syrup (HFCS) consumption. Data are expressed as the means ± SEs. The asterisks indicate statistically significant differences (*p < 0.05, **p < 0.01, ***p < 0.001; n = 5–8, Student’s *t*-test at each age bracket).

We examined the effect of food texture in mice consuming tap water or HFCS. Body weight and total calorie intake (*i*.*e*., sum of diet and HFCS) were not affected by food texture irrespective of HFCS administration (Fig 2A and 2B). However, the ratio of energy intake from both solid food and HFCS water was remarkably affected by food texture. When mice were provided HFCS, the volume of fluid consumed was significantly increased in the hard food group compared to the soft food group, whereas the intake of tap water did not differ between the two groups (Fig 2C). Meanwhile, when mice drank HFCS, calories from food were lower in the hard food group than in the soft food group (Fig 2D).

### Involvement of μ-opioid in the increase of HFCS consumption in mice fed hard food

**Fig 3:**
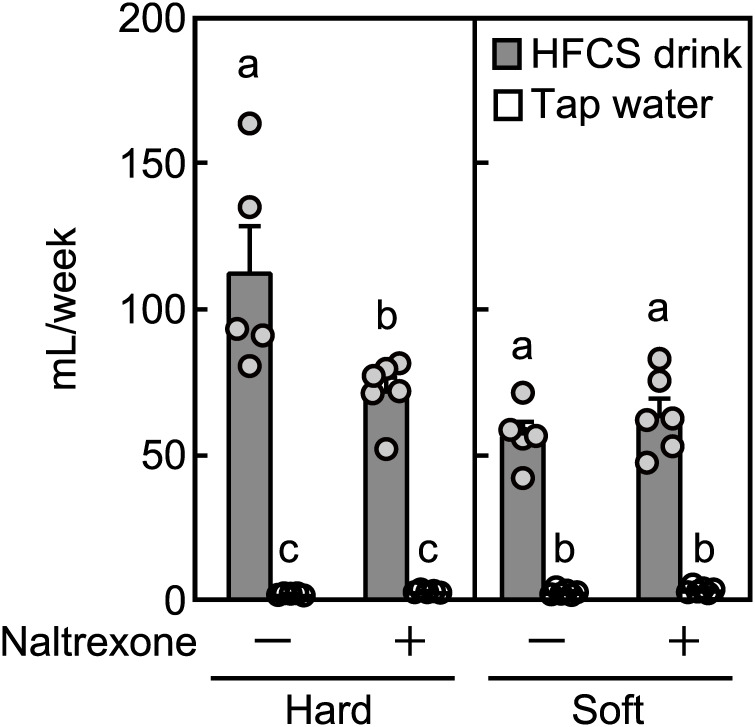
Evaluation of μ-opioid with regard to the preference for high-fructose corn syrup (HFCS) in mice. Five-week-old mice were injected with 10 mg/kg naltrexone twice/day for 7 days. Food and drink intakes were measured. Data are expressed as the means ± SEs. The different letters indicate statistically significant differences (*p* < 0.05; n = 5–6, two-way ANOVA, Tukey-Kramer’s post-hoc testing).

To examine the involvement of μ-opioid in the difference in HFCS intake between the hard and soft food groups, the μ-opioid receptor antagonist naltrexone was subcutaneously injected twice per day for 7 days. Inhibition of the μ-opioid receptor by naltrexone abrogated the increase in HFCS consumption in the hard food group (Fig 3).

### Effect of food texture on glucose and insulin tolerance in mice consuming HFCS

**Fig 4:**
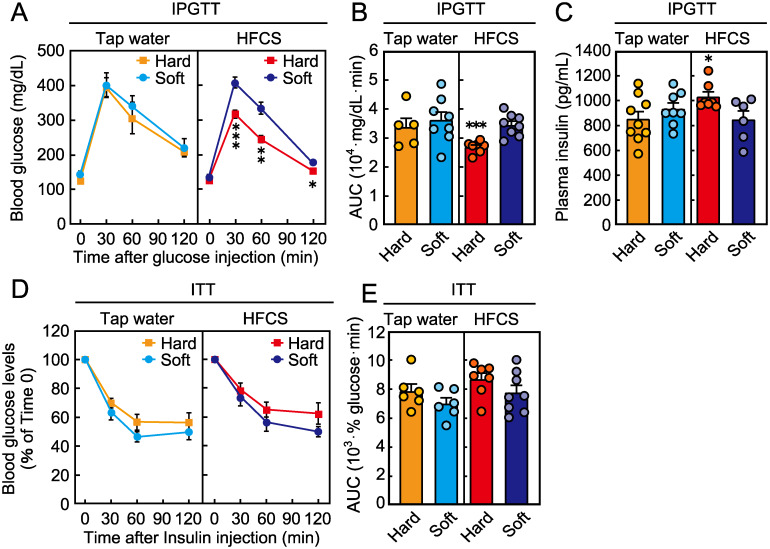
Effect of food texture on glucose and insulin metabolism. (A) Blood glucose levels and (B) area under the curve for the intraperitoneal glucose tolerance test (IPGTT) in mice at 16 weeks of age. (C) Plasma insulin levels after stimulation with glucose for 15 min. (D) Blood glucose levels and (E) area under the curve for the insulin tolerance test (ITT) in mice at 17 weeks of age. Data are expressed as the means ± SEs. The asterisks indicate statistically significant differences (*p < 0.05, **p < 0.01, ***p < 0.001; n = 5–9, Student’s *t*-test at each time point).

Next, we evaluated the effect of food texture on glucose metabolism. When mice were provided tap water, glucose tolerance was not affected by food texture (Fig 4A). In contrast, when mice were provided HFCS, glucose levels according to an intraperitoneal glucose tolerance test (IPGTT) were lower in the hard food group at 30, 60, and 120 min after glucose challenge. These data were supported by the AUC of the IPGTT (Fig 4B). Insulin concentrations after glucose stimulation were significantly higher in the hard food group than in the soft food group only when mice were administered HFCS (Fig 4C). In contrast, food texture had no effect on insulin resistance according to an insulin tolerance test (ITT), irrespective of HFCS intake (Fig 4D and 4E).

### Effect of food texture on pancreatic β-cell mass and insulin levels

**Fig 5:**
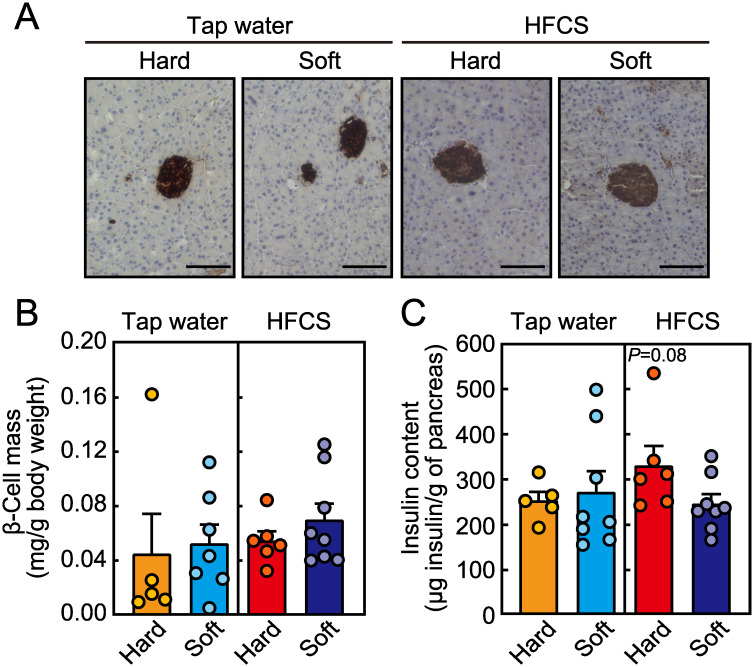
Effect of food texture on pancreatic β-cell mass and insulin contents. (A) Representative image of insulin-positive β-cells. Comparison of (B) β-cell mass and (C) insulin content in the pancreas. Data are expressed as the means ± SEs. (n = 5–8, Student’s *t*-test).

To elucidate the mechanism underlying the effect of food texture on pancreatic β-cell function, we evaluated the insulin contents in the pancreas. Food texture did not affect total pancreatic tissue weight (Table 1). In addition, pancreatic β-cell mass was not affected by food texture, irrespective of HFCS consumption (Fig 5A and 5B). In contrast, hard food tended to increase insulin contents only when HFCS was provided (Fig 5C).

**Table 1:** Relative organ weights (% of body weight).

	Tap water		HFCS	
	Hard	Soft	Hard	Soft
Visceral WAT				
Mesenteric	0.66±0.08	0.68±0.12	0.56±0.07	0.50±0.06
Perirenal/retroperitoneal	0.42±0.05	0.52±0.12	0.39±0.05	0.36±0.05
Epididymal	1.08±0.09	1.20±0.21	1.09±0.08	0.98±0.09
Subcutaneous WAT				
Inguinal	0.99±0.12	0.63±0.15	0.73±0.10	0.69±0.06
Skeletal muscle				
Quadriceps	1.35±0.05	1.25±0.05	1.31±0.20	1.14±0.06
Hamstrings	2.71±0.03	2.69±0.14	2.31±0.17	2.53±0.07
Masseter	0.60±0.04	0.57±0.03	0.57±0.03	0.52±0.04
Salivary gland				
Submandibular	0.47±0.02	0.46±0.01	0.47±0.01	0.48±0.01
Sublingual	0.05±0.002	0.06±0.003*	0.06±0.004	0.08±0.004*
Parotid	0.37±0.02	0.37±0.02	0.43±0.02	0.42±0.03
Others				
Liver	3.88±0.18	4.50±0.47	4.01±0.13	4.10±0.37
Kidney	1.35±0.06	1.37±0.07	1.26±0.06	1.28±0.03
Pancreas	1.07±0.07	0.99±0.06	1.03±0.07	1.10±0.01

Data are expressed as the means ± SEs (*p<0.05, ^#^*p*<0.10, n = 5–8, Student’s *t*-test). WAT, white adipose tissue

### Effect of food texture on liver and kidney function

**Fig 6:**
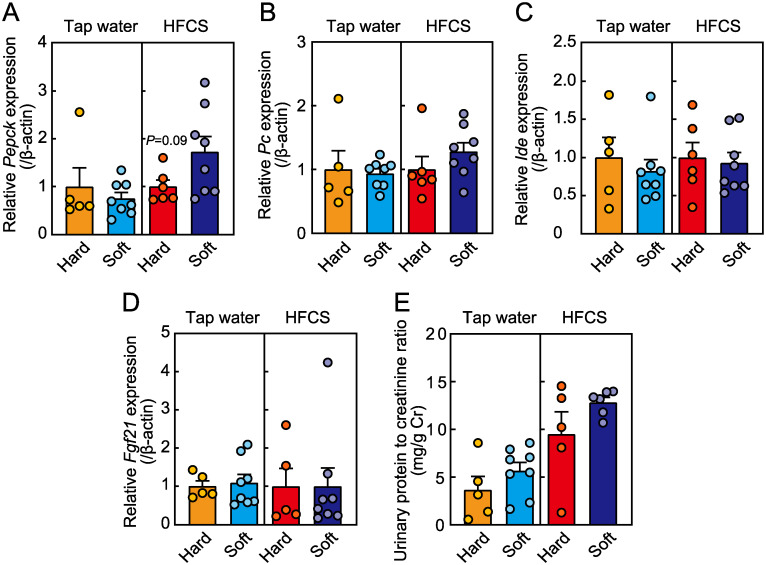
Expression profiles of genes regulating blood glucose in the liver. Expression of (A) *phosphoenolpyruvate carboxykinase* (*Pepck*), (B) *pyruvate carboxylase* (*Pc*), (C) *fibroblast growth factor 21* (*Fgf21*), and (D) *insulin degrading enzyme* (*Ide*) in the liver. Target gene expression was normalised to the expression of *β-actin*. (E) Urine protein-to-creatinine ratio. Data are expressed as the means ± SEs. (n = 5–8, Student’s *t*-test).

Expression of the liver gluconeogenesis-related gene *phosphoenolpyruvate carboxykinase* (*Pepck*), but not *pyruvate carboxylase* (*Pc*), tended to be lower in the hard food group than in the soft food group (Fig 6A and 6B). However, food texture did not affect the expression of *fibroblast growth factor 21* (*Fgf21*) and *insulin degrading enzyme* (*Ide*) (Fig 6C and 6D). Liver weight, as well as triglyceride and cholesterol levels, were not affected by food texture irrespective of HFCS (Tables 1 and 2). Due to increased consumption of fluid in the hard food group consuming HFCS, we examined the kidney function. The urine albumin-to-creatinine ratio, a marker of kidney function, was not affected by food texture even when mice consumed HFCS (Fig 6E).

**Table 2:** Biochemical parameters of the plasma and liver.

	Tap water		HFCS	
	Hard	Soft	Hard	Soft
Plasma				
Triglyceride (mg/dL)	48.92±7.36	55.07±6.09	44.25±2.09	46.00±3.28
Cholesterol (mg/dL)	76.17±11.27	72.64±8.38	96.77±2.39	96.56±5.77
Adiponectin (relative value)	1.00±0.29	1.22±0.56	1.00±0.17	1.28±0.43
Liver				
Triglyceride (mg/g liver)	3.07±0.96	4.65±0.60	5.03±1.18	2.89±0.71
Cholesterol (mg/g liver)	2.59±0.56	3.08±0.34	1.75±0.06	1.61±0.14

Data are expressed as the means ± SEs (n = 5–8, Student’s *t*-test). Plasma and liver samples were analysed after dissection.

### Effect of food texture on tissue weight and plasma lipid and adiponectin levels

Adipose and muscle tissue weights were not affected by the hardness of food (Table 1). The sublingual grand, but not the submandibular or parotid grand, was heavier in the soft food group than in the hard food group irrespective of HFCS consumption. Food texture did not affect plasma adiponectin, triacylglycerol, or cholesterol levels (Table 2).

## Discussion

Diet-related factors are assumed to affect glucose metabolism. Excess total energy intake and nutritional composition (*e*.*g*., high-fat diet) are critical determinants of glucose metabolism disorders. The hardness of food [5–7] and the consumption of HFCS [13, 14] are thought to be involved in the regulation of glucose metabolism. However, the combined effects of these dietary factors on glucose metabolism is largely unknown. In the present study, we investigated the effects of ingestion of food with either hard or soft texture, and found that hard food ameliorated glucose tolerance in mice consuming HFCS.

We found that blood glucose levels were lower in the hard food group, despite the increased consumption of HFCS, than in the soft food group. This result is unexpected and surprising because an increased HFCS intake has been proposed to be associated with impaired glucose tolerance [11]. Mice receiving hard food had significantly lower glucose levels soon after glucose challenge, suggesting enhanced β-cell function [23], whereas the plasma insulin concentrations after glucose stimulation were higher in the hard food group than in the soft food group. The insulin content in the pancreas, which is important for blood glucose control [24, 25], tended to be higher in mice receiving hard food. These differences in glucose tolerance between mice fed hard- or soft food may be related to lower levels of *Pepck* expression in the hard food group, which suggests the suppression of gluconeogenesis. However, insulin sensitivity was not different between the two groups, and this contribution to glucose tolerance thus seems to be minor. Unlike previously reported effects [6], adiponectin levels were not affected by food texture in the present study. Together, these results suggest that the amelioration of glucose tolerance in the hard food group consuming HFCS is due to increased pancreatic β-cell function.

In the present study, a difference in food texture alone did not affect glucose intolerance. Previous studies have reported an increase in glucose tolerance in rodents fed a pellet diet compared with a soft powder diet. NSY mice, which spontaneously develop diabetes, become overweight and exhibit glucose intolerance when fed a soft powder diet [7]. Although rats fed a powder diet did not gain body weight, they exhibited increased insulin resistance and glucose intolerance in parallel with increased insulin levels [26]. In contrast, decreased glucose tolerance due to HFCS or fructose intake has been also reported. The consumption of 4.2% HFCS with a high-fat diet increases body weight and impairs glucose tolerance in mice [16]. Glucose intolerance and abnormal β-cell morphology occurs in rats drinking 7% fructose, without lipid accumulation in the liver [27]. In the present study, we observed the amelioration of glucose tolerance in the hard food group consuming 4.2% HFCS, without any changes in the lipid levels in the liver or blood.

The hardness of food did not affect food intake, body weight, body fat mass, or plasma lipid levels in the present study. Although glucose homeostasis is closely related to obesity [28], based on these results the regulation of glucose homeostasis is independent to obesity. In addition, our results differ from previous reports where a soft diet has resulted in overeating and obesity [6], or that feeding efficiency (*i*.*e*., body weight gain/energy intake) is affected by food texture [7, 8]. Brain hypothalamus function is involved in overeating and feeding efficiency [29], as well as sensing food texture [30]. Therefore, these discrepancies in overeating and feed efficiency may be derived from the combined effects of food texture and food components such as fats on the hypothalamus.

The increase in HFCS consumption in the hard food group was abolished when mice were treated with the μ-opioid receptor antagonist naltrexone. β-Endorphin is an opioid hormone that influences the brain’s reward system [31] and is activated by HFCS in the hypothalamus [32]. Therefore, the combination of HFCS consumption and a hard food diet may strongly activate the brain’s reward system, causing addiction. β-Endorphin may contribute to the regulation of glucose metabolism by stimulating insulin secretion from pancreatic β-cells [33, 34], although this remains controversial [35]. Therefore, β-endorphin may be involved in the observed improvement in β-cell function and glucose tolerance.

Fructose feeding (fructose or fructose plus a high-fat diet but not a high-fat diet alone) accelerates gastric emptying [36–38], whereas solid-diet feeding delays it [39] affecting the secretion of gut hormones (*e*.*g*., gastric inhibitory polypeptide, glucagon-like peptide-1, cholecystokinin, peptide YY, ghrelin, and leptin) [40, 41]. These gut hormones affect the brainstem, including the hypothalamus, to modulate appetite and eating behavior [42]. A limitation of this study is that we did not address the effects of gut hormones, although we found differences in the glucose tolerance test with intraperitoneal injection of glucose. Several responses to glucose, such as in the gut and brain, could not be observed by this method owing to the bypassing of the gut. Future studies concerning the involvement of the gut are required to uncover the complete physiological effects of a hard food with HFCS as well as the detailed mechanisms underlying the enhancement of glucose tolerance and β-cell function.

In conclusion, the hardness of daily food affects chewing habits. In addition to food composition, the physical texture of food may also impact energy metabolism by affecting chewing habits. This is the first study to demonstrate the interactive effects of food texture and HFCS consumption on glucose tolerance. The consumption of low doses of HFCS may partly contribute to enhance β-cell function, which may explain why people in Western countries have a more robust β-cell function than those in Asian countries. Factors such as the consumption of HFCS may play a crucial role in the observed effects of food texture on the development of metabolic disorders.

## Supporting information

S1 Raw images (PDF)
