# Supplementary figures and images for: Food texture affects glucose tolerance by altering pancreatic β-cell function in mice consuming high-fructose corn syrup
Source: PLoS One. 2020 May 29;15(5):e0233797. doi: 10.1371/journal.pone.0233797 (PMC7259500; doi:10.1371/journal.pone.0233797)

Table 2 Plasma adiponectin levels

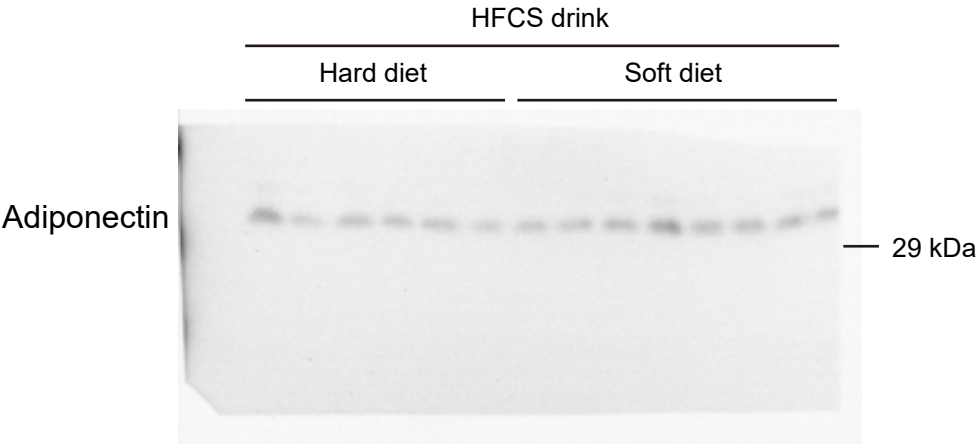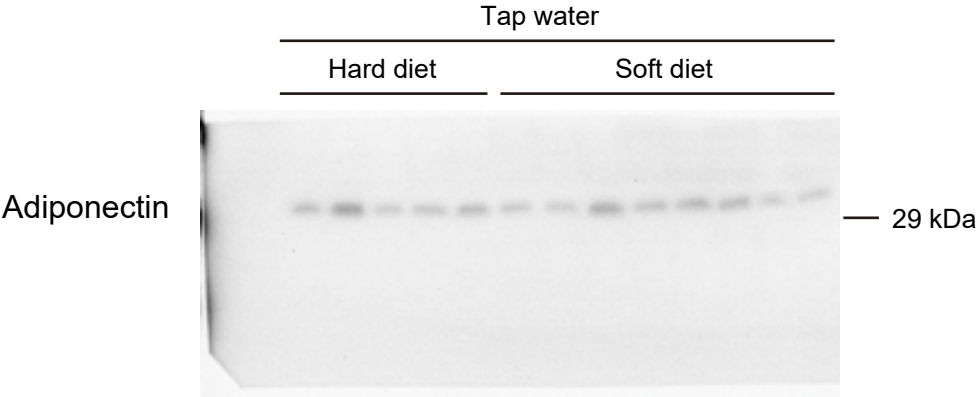

Supplement: S1 Raw images — (PDF) [file pone.0233797.s001.pdf]
